# Beyond clinical closure: functional skin barrier recovery to prevent diabetic foot ulcer recurrence: a narrative review

**DOI:** 10.3389/fendo.2026.1918476

**Published:** 2026-08-10

**Authors:** Yulan Cai, Feng Zeng, Chuyu Liu

**Affiliations:** 1 Department of Endocrinology, the Second Affiliated Hospital of Zunyi Medical University, Zunyi, Guizhou, China; 2 Department of Endocrinology and Metabolism, Affiliated Hospital of Zunyi Medical University, Zunyi, Guizhou, China; 3 Department of Thyroid and Breast Surgery, the Second Affiliated Hospital of Zunyi Medical University, Zunyi, Guizhou, China

**Keywords:** diabetic foot ulcer, functional closure, recurrence, skin barrier, transepidermal water loss

## Abstract

Diabetic foot ulcer (DFU) recurrence remains common after apparent wound healing, partly because conventional endpoints define healing as complete epithelialization without drainage rather than as durable recovery of skin function. This narrative review proposes functional closure as a clinically useful extension of current DFU healing assessment. Functional closure refers to a post-healing state in which the regenerated ulcer site has recovered sufficient epidermal barrier competence to limit transepidermal water loss (TEWL), maintain stratum corneum hydration, tolerate minor mechanical trauma, and reduce microbial entry. Diabetes-related epidermal dysfunction, autonomic neuropathy, anhidrosis, xerosis, fissuring, altered lipid metabolism, impaired perfusion, and repetitive plantar loading may leave clinically closed ulcer sites biologically fragile. Emerging evidence linking elevated TEWL at recently closed chronic wounds and DFU sites with subsequent recurrence supports the incorporation of barrier assessment into post-closure risk evaluation. However, existing moisturizer and barrier-supporting studies have mainly demonstrated improvements in xerosis, hydration, and visible skin condition, and have not yet established a reduction in recurrent DFU. Post-healing DFU care should therefore move beyond the binary distinction between an open and a closed wound toward active maintenance of durable remission. Future trials should test targeted barrier-repair strategies in risk-enriched patients with high TEWL, poor hydration, fissuring, callus, fragile scar skin, or other signs of incomplete functional closure, while integrating barrier assessment with offloading, plantar pressure evaluation, temperature monitoring, vascular assessment, infection surveillance, and professional foot care. This framework may help shift DFU management from treating recurrent breakdown to preserving long-term barrier-competent healing. Importantly, functional closure is also framed as an immunologically competent barrier state, in which the healed surface limits persistent irritant- and microbe-driven activation of innate immune pathways rather than merely appearing epithelialized or hydrated. TEWL should be interpreted as one component of barrier recovery rather than as a single surrogate definition of functional closure, and the proposed framework remains a conceptual model requiring prospective validation.

## Background

Diabetic foot ulceration remains one of the most serious lower-extremity complications of diabetes. A diabetic foot ulcer (DFU) is frequently accompanied by infection, hospitalization, impaired mobility, reduced quality of life, amputation risk, and excess mortality. Despite advances in multidisciplinary wound care, offloading, infection control, vascular assessment, and metabolic management, many ulcers remain difficult to heal. Contemporary evidence suggests that only approximately 30%-40% of DFUs heal within 12 weeks under current standards of care (1). More importantly, apparent closure does not end the clinical risk; patients remain vulnerable to recurrent tissue breakdown, infection, limb loss, and premature mortality.

Recurrence is a defining feature of DFU rather than an unusual event after healing. Recurrence after ulcer closure has been estimated at approximately 42% at 1 year and 65% at 5 years in a recent clinical review (1). The International Working Group on the Diabetic Foot (IWGDF) similarly emphasizes recurrence rates of approximately 40% within 1 year and 65% within 3 years (2). A systematic review and meta-analysis estimated a pooled global recurrence rate of 22.1% per person-year, and long-term cohorts have reported cumulative recurrence approaching 70% during extended follow-up (3, 4). These data support the view that recurrence is a chronic and cumulative process, not only an early post-healing complication.

This evidence has led to an important conceptual shift: a healed DFU should be regarded as being in remission rather than cured. The biological, biomechanical, and vascular conditions that caused the initial ulcer often persist after epithelial closure. Loss of protective sensation, peripheral artery disease, deformity, repetitive mechanical stress, callus, impaired self-care, and tissue fragility may all remain present (2, 5). The post-closure period should therefore be treated as an active phase of disease management, with the goal of extending ulcer-free time rather than merely documenting closure.

Current prevention strategies largely follow this remission-based model. The IWGDF prevention guideline recommends regular risk stratification, screening for loss of protective sensation and peripheral artery disease, structured education, avoidance of barefoot walking or unsuitable footwear, treatment of pre-ulcerative lesions, and integrated professional foot care (2). These interventions are indispensable, particularly because recurrent ulceration is often driven by neuropathy, pressure overload, deformity, callus, and inadequate offloading. However, most preventive approaches focus on mechanical, vascular, infectious, footwear-related, and behavioral risk. The biological quality of the newly closed skin surface has received comparatively less attention.

This gap is clinically relevant because conventional wound closure is largely morphological. In trials and routine practice, a DFU is generally considered healed when the surface is completely epithelialized, free of drainage, and clinically closed. This definition is practical, but it does not show whether the regenerated epidermis has recovered water-barrier function, hydration balance, mechanical resilience, or resistance to fissuring and microbial penetration. Recent work in chronic wounds and the NIDDK Diabetic Foot Consortium TEWL Study suggests that elevated TEWL at clinically closed wound sites may identify incomplete functional closure and higher recurrence vulnerability (6, 7).

In this review, we argue that post-healing DFU care should move beyond the binary distinction between an open and a closed wound. The central question is not only whether the ulcer surface has epithelialized, but whether the newly closed site has recovered enough barrier competence to remain closed under daily mechanical, microbial, and environmental stress. We use the term functional closure to describe this more durable state of healing. On this basis, we examine the biological vulnerability of diabetic skin, the role of TEWL and complementary skin assessments as post-closure risk markers, and the rationale for testing barrier-repair strategies in recurrence-prevention trials.

## Evidence acquisition

This narrative review was developed through a targeted literature search and expert synthesis. PubMed, Web of Science, and Scopus were searched from inception to 6 June 2026. Search terms included “diabetic foot ulcer”, “recurrence”, “functional closure”, “transepidermal water loss”, “skin barrier”, “xerosis”, “moisturizer”, “barrier repair”, “plantar pressure”, “plantar temperature”, “temperature monitoring”, and “digital foot monitoring”. We prioritized international guidelines, clinical reviews, prospective cohort studies, randomized trials, systematic reviews and meta-analyses, prognostic biomarker studies, and biophysical studies relevant to diabetic skin barrier function or post-closure recurrence prevention. Additional publications were identified from the reference lists of key articles. Because the purpose was to develop a clinically oriented conceptual framework and identify translational gaps rather than estimate pooled effects, no formal risk-of-bias assessment or meta-analysis was performed. This article should therefore be interpreted as a narrative review, not a systematic review or meta-analysis.

For the interventional evidence on foot skin care and barrier repair, the implicit PICO logic was as follows: the population comprised people with diabetes, diabetic foot xerosis, at-risk feet, or, when available, patients after DFU closure; the interventions included topical moisturizers, humectants, occlusive protectants, keratolytic-hydrating agents, and lipid-repair formulations; comparators included vehicle cream, base cream, standard emollient, usual foot care, or another moisturizer; and outcomes included xerosis severity, fissures, stratum corneum hydration, TEWL, skin-condition scores, tolerability, adherence, DFU incidence, and recurrent ulceration. We did not include systemic therapies, products intended only for open infected wounds, surgical dressings, or animal-only biomaterial studies as primary interventional evidence for post-closure barrier repair. Because these studies differed substantially in population risk, product composition, comparator choice, follow-up duration, outcome definition, and control of confounding factors such as offloading, neuropathy, peripheral artery disease, and footwear, the intervention evidence was synthesized narratively rather than as a pooled effect estimate.

## Clinical closure is not functional closure: redefining the endpoint of diabetic foot healing

In clinical trials and routine wound care, DFU healing is most often defined by visible wound closure. Regulatory guidance for chronic cutaneous ulcer trials has commonly defined complete wound closure as skin re-epithelialization without drainage or dressing requirements, confirmed at two consecutive study visits (8). This definition has practical strengths: it is observable, clinically meaningful, and reasonably reproducible. Yet it remains a morphological endpoint. It confirms that the wound surface appears closed, but it does not determine whether the regenerated epidermis has recovered the physiological properties of intact skin.

The limitations of closure-based endpoints have long been recognized in DFU research. The Los Angeles DFCon consensus emphasized complete wound closure as a central endpoint in DFU trials, while acknowledging that different therapies are commonly judged against the same visible outcome (9). Early wound area reduction is also useful, as the percentage reduction in DFU area over 4 weeks predicts later healing (10). Even so, both wound area reduction and visible closure remain measures of surface repair. They do not assess permeability barrier function, resistance to friction, or the capacity to withstand repetitive plantar stress after closure.

This distinction matters in DFU because a recently closed ulcer is rapidly re-exposed to loading, shear, footwear friction, callus formation, altered sweating, and microbial contamination. In patients with neuropathy, these stresses may occur without protective pain perception. In patients with peripheral artery disease, microvascular dysfunction, or impaired tissue repair, the newly healed site may remodel slowly and remain fragile. The closed surface of a previously ulcerated foot should therefore not be assumed to function like adjacent non-ulcerated skin simply because it appears epithelialized.

The concept of functional closure provides a more biologically complete framework for durable DFU healing. Functional closure means that the wound has not only achieved surface continuity, but has also regained enough barrier integrity to limit water loss, maintain stratum corneum hydration, resist minor mechanical injury, and reduce penetration of irritants or microorganisms. Re-epithelialization remains essential, but it is not sufficient. In this model, the quality of the newly formed epidermal barrier becomes part of the healing endpoint, and barrier failure may precede visible reopening in some recurrent ulcers.

For clarity, TEWL is not proposed as the definition of functional closure. Rather, functional closure is a multidimensional construct that includes structural continuity, water-barrier integrity, stratum corneum hydration, local skin quality, resistance to minor mechanical stress, and limitation of unnecessary irritant- or microbe-driven immune activation. TEWL is currently the most direct candidate measure of epidermal water-barrier integrity, but it should be interpreted together with clinical skin assessment and mechanical risk factors (**Table 1**).

**Table 1:** Proposed components and assessment domains of functional closure after diabetic foot ulcer healing.

Component	Possible clinical assessment	Evidence status
Structural continuity	Complete epithelialization, absence of exudate, and no macroscopic reopening	Established clinical healing criterion
Water-barrier integrity	TEWL at the healed site, preferably under standardized environmental conditions and with contralateral or periwound comparison	Direct DFU recurrence evidence is emerging; clinically actionable thresholds are not yet standardized
Stratum corneum hydration	Corneometry or low-cost hydration assessment when available; clinical dryness assessment when instruments are unavailable	Supported mainly by indirect diabetic skin and xerosis evidence
Local skin quality	Xerosis, fissures, callus, scar fragility, maceration, erythema, and standardized foot photography	Clinically plausible and partly supported by diabetic foot skin studies
Mechanical tolerance	Plantar pressure assessment, footwear fit, offloading adherence, callus recurrence, and deformity assessment	Established relevance to recurrence prevention, but not specific to skin barrier recovery
Immunological competence	Absence of persistent irritation, infection-prone skin breaks, maceration, or inflammatory signs; future studies may include microbial and inflammatory markers	Hypothesis-generating; extrapolated from skin barrier and inflammation biology

This table is intended to clarify the proposed construct of functional closure. It does not imply that all components have validated thresholds or that any single marker, including TEWL, can by itself define functional closure.

**Figure 1:**
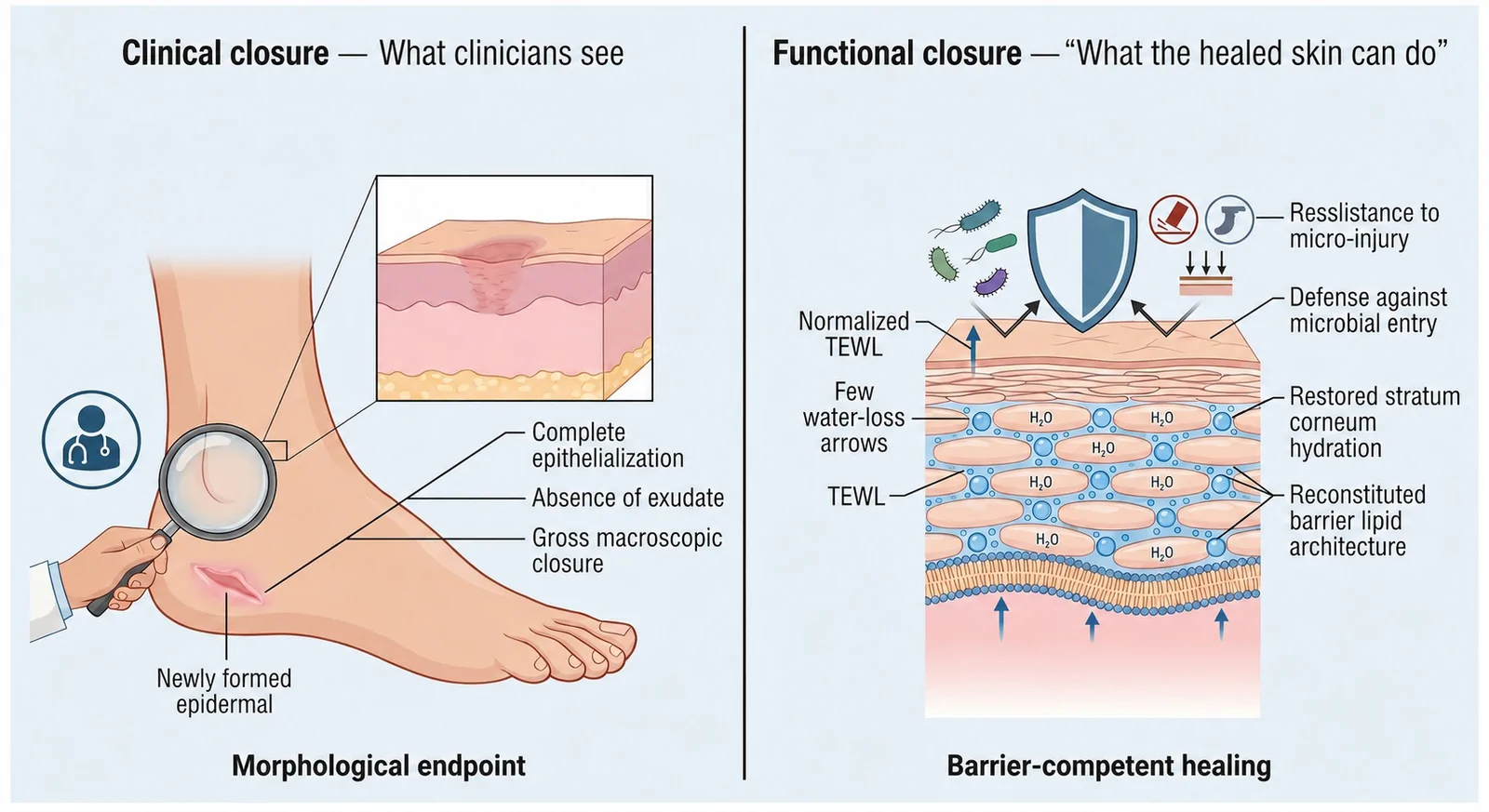
Clinical closure versus functional closure in diabetic foot ulcer healing. Clinical closure represents what can be observed on routine inspection: complete epithelialization, absence of exudate, and gross macroscopic closure of the wound surface. These features are necessary clinical milestones, but they primarily define a morphological endpoint. Functional closure refers to what the healed skin can do after apparent wound closure. It requires restoration of key protective properties of the regenerated epidermis, including normalized transepidermal water loss (TEWL), restored stratum corneum hydration, reconstituted barrier lipid architecture, resistance to minor mechanical injury, and defense against microbial entry. This distinction emphasizes that an apparently healed DFU may remain biologically vulnerable if the closed surface has not regained adequate barrier competence. Because functional closure cannot be judged by visual inspection alone, post-closure assessment may require a layered approach: confirmation of epithelial continuity, structured evaluation of xerosis, fissuring, callus and scar fragility, and objective barrier testing when available. TEWL is one candidate measure of this functional dimension.

Thus, clinical closure represents a necessary visible milestone, whereas functional closure reflects the biological quality and durability of the healed surface. This conceptual distinction is illustrated in **Figure 1**.

## Skin barrier dysfunction in diabetes: the biological basis of post-closure vulnerability

The vulnerability of a recently closed diabetic foot ulcer cannot be explained solely by the local history of ulceration. Diabetes is associated with systemic alterations in epidermal structure and function that may affect both previously ulcerated and non-ulcerated skin. In type 2 diabetes, experimental and clinical evidence indicates reduced stratum corneum hydration, increased epidermal permeability, delayed permeability barrier recovery, and altered skin surface pH. These abnormalities may contribute to cutaneous inflammation, pruritus, dryness, and susceptibility to minor trauma. Therefore, a newly epithelialized DFU surface heals within a host environment in which epidermal homeostasis may already be compromised (11).

Several mechanisms may contribute to this barrier dysfunction. Chronic hyperglycemia may affect keratinocyte proliferation and differentiation, alter epidermal lipid metabolism, promote advanced glycation end-product accumulation, and impair the biomechanical properties of skin. Systemic low-grade inflammation may also disturb the lipid organization required for an intact stratum corneum barrier. Recent experimental work in diabetes has emphasized that inflammation and altered ceramide metabolism can attenuate epidermal permeability barrier formation. Although much of this mechanistic evidence has been generated outside the DFU setting, it provides a biologically plausible explanation for why diabetic skin may remain structurally and functionally fragile even after visible wound closure (11, 12).

Clinical biophysical studies support the presence of measurable skin barrier abnormalities in diabetes. In a study evaluating morphological and structural skin alterations in individuals with diabetes, diabetic skin showed higher transepidermal water loss values than healthy skin, suggesting impaired barrier integrity. The same study also reported differences in mechanical and morphological skin characteristics, indicating that diabetic skin is not only metabolically abnormal but also structurally altered. These findings are relevant to DFU recurrence because a healed wound site must tolerate repetitive loading, shear stress, and footwear-related friction; if the surrounding skin is already mechanically altered or barrier-compromised, the threshold for breakdown may be reduced (13).

Xerosis is one of the most common clinical manifestations of diabetic skin barrier dysfunction. It is characterized by dryness, scaling, flaking, roughness, and, in more severe cases, fissuring. In patients with type 2 diabetes, xerosis is highly prevalent and frequently affects the lower limbs and feet. This condition should not be regarded as merely cosmetic. Dry and fissured skin may create entry points for irritants and microorganisms, increase local inflammation, and predispose to superficial injury. For a patient with a recently healed DFU, persistent xerosis around the healed site or on adjacent plantar surfaces may therefore represent a clinically visible marker of residual vulnerability (14).

The clinical relevance of foot dryness is supported by studies linking moisture status and future or prevalent ulceration. In a cross-sectional study of 379 individuals with diabetes, abnormal foot moisture status assessed using the Neuropad visual test was associated with the presence of foot ulceration, even after accounting for measures of neuropathy. A subsequent prospective multicenter study found that dryness of foot skin assessed by the visual indicator test predicted the development of DFU and could be incorporated into screening strategies for the foot at risk. These findings suggest that dryness and sudomotor dysfunction are not only dermatologic findings, but clinically meaningful markers of ulcer susceptibility (15, 16).

The diabetic foot is particularly susceptible to xerosis and fissuring because cutaneous barrier dysfunction is compounded by neuropathy. Peripheral autonomic neuropathy can reduce sweating, leading to anhidrosis, dryness, scaling, and fissures. At the same time, sensory neuropathy reduces protective perception, allowing repetitive friction or minor skin injury to progress without early warning symptoms. A case-control study assessing water evaporation as a measure of permeability barrier function found that diabetic skin barrier status was related to both sensorimotor and autonomic neuropathy. This supports the view that impaired neural regulation and barrier dysfunction are biologically linked rather than independent features of diabetes (17).

The plantar surface adds another layer of risk. Dry diabetic foot skin may be stiffer and more prone to superficial fissuring than dry skin in non-diabetic individuals. In an exploratory study comparing xerotic foot skin between diabetic and non-diabetic participants, the diabetic group had a substantially higher number of superficial fissures and greater skin stiffness. These findings are important because fissures may act as micro-wounds, particularly in regions exposed to pressure and shear. After DFU closure, even small fissures near the original ulcer site or within high-pressure plantar regions may serve as early points of recurrent tissue breakdown (18).

Not all studies show uniform abnormalities in epidermal barrier parameters across all diabetic populations. For example, a prospective case-control study found no overall difference in TEWL and skin hydration between individuals with diabetes and non-diabetic controls, although reduced skin hydration within the diabetic group was associated with uncontrolled fasting blood glucose and peripheral neuropathy. Similarly, a recent observational study in children and adolescents with type 1 diabetes evaluated TEWL and epidermal hydration as markers of epidermal barrier function and found that barrier parameters varied with disease-related factors. These findings indicate that skin barrier dysfunction in diabetes is likely influenced by age, disease duration, metabolic control, neuropathy, adiposity, and the presence of complications, rather than by diabetes diagnosis alone (19, 20).

## Barrier-inflammation crosstalk in post-closure vulnerability

A defective post-closure barrier is not only a biophysical problem. Increased permeability, reflected in part by elevated TEWL, may allow irritants, microbial products, and damage-associated signals to persist at or penetrate into the newly regenerated epidermis. Keratinocytes, Langerhans cells, macrophages, and other resident or recruited immune cells express pattern-recognition receptors, including Toll-like receptors, that can sense pathogen-associated and damage-associated molecular patterns. Activation of these pathways can amplify NF-kB-dependent inflammatory programs and increase the production of cytokines and chemokines such as interleukin-1 beta, tumor necrosis factor-alpha, interleukin-6, and CXCL8/IL-8 (21, 22).

This inflammatory activation may create a self-reinforcing barrier-inflammation loop. Local cytokines, oxidative stress, proteases, and persistent cellular stress can interfere with keratinocyte differentiation, lamellar body secretion, antimicrobial peptide balance, and the organization of stratum corneum lipids, including ceramides, cholesterol, and free fatty acids. Conversely, impaired lipid architecture and low stratum corneum hydration further increase permeability and microbial access. In diabetes, hyperglycemia, altered lipid metabolism, microvascular impairment, and neuropathy-related anhidrosis may lower the threshold for this cycle to persist after clinical closure (11, 12, 23, 24).

Functional closure should therefore be considered not only a hydration- or mechanics-based endpoint, but also an immunologically competent endpoint. A barrier-competent healed DFU surface should limit unnecessary innate immune stimulation, reduce subclinical irritant or microbial challenge, and permit resolution of low-grade inflammation. From this perspective, barrier-repair strategies are relevant not simply because they moisturize dry skin, but because they may help interrupt the cycle of barrier leakiness, microbial or irritant exposure, and chronic inflammatory activation. Direct DFU-specific evidence for this immunological benefit is still limited, and this remains an important target for future mechanistic and clinical studies.

Post-closure vulnerability is therefore unlikely to be confined to the scar or local wound bed. It is also shaped by the broader biology of diabetic skin. Reduced stratum corneum hydration, delayed barrier recovery, altered lipid composition, xerosis, fissuring, neuropathy-related anhidrosis, and increased mechanical stiffness may interact to produce a fragile surface after clinical healing. This systemic barrier vulnerability supports post-closure assessment of skin quality and justifies further investigation of barrier-repair strategies as part of recurrence prevention. In this context, the recently closed DFU should be viewed not as normal skin restored to baseline, but as a vulnerable surface embedded within a metabolically, neurologically, and biomechanically compromised cutaneous environment.

**Figure 2:**
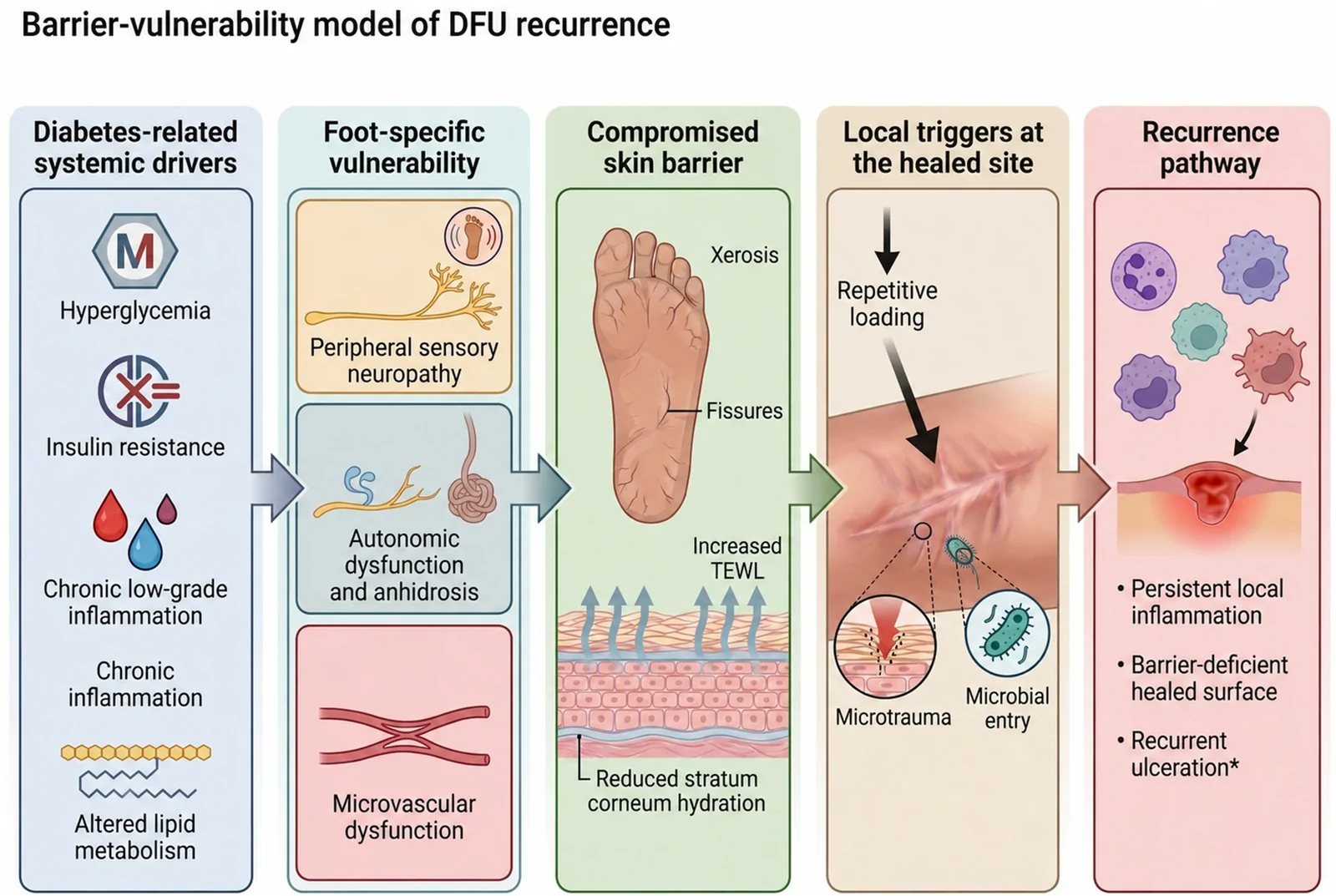
Barrier-vulnerability model linking diabetes-associated skin barrier dysfunction to diabetic foot ulcer recurrence. Diabetes may compromise the durability of diabetic foot ulcer (DFU) closure through systemic metabolic and inflammatory disturbances, including hyperglycemia, insulin resistance, chronic low-grade inflammation, and altered lipid metabolism. These systemic drivers interact with foot-specific vulnerabilities, such as peripheral sensory neuropathy, autonomic dysfunction with anhidrosis, and microvascular dysfunction. Together, these factors may impair epidermal barrier integrity, leading to xerosis, fissuring, increased transepidermal water loss (TEWL), and reduced stratum corneum hydration. At the recently healed ulcer site, repetitive loading and shear stress may induce microtrauma, while barrier disruption may facilitate microbial entry. This barrier-deficient surface can sustain local inflammation and increase susceptibility to recurrent ulceration, particularly at previously ulcerated or high-pressure plantar sites. This model supports post-closure assessment of skin barrier quality as a component of recurrence-risk evaluation. The model also highlights that a leaky post-closure barrier may sustain innate immune activation through pattern-recognition pathways, linking TEWL-defined barrier failure to persistent inflammatory risk.

These mechanisms can be integrated into a barrier-vulnerability model of DFU recurrence. In this model, diabetes-associated neuropathy, anhidrosis, and microvascular dysfunction converge to impair epidermal barrier integrity. The resulting xerosis, fissuring, and increased transepidermal water loss may create a fragile post-closure surface that is susceptible to microtrauma, microbial entry, and persistent low-grade inflammation. Once this fragile surface is exposed to repetitive mechanical stress and microbial challenge, recurrent ulceration may develop at the original wound site or at adjacent high-risk plantar regions. This proposed pathway is summarized in **Figure 2**.

## TEWL as a biomarker of post-closure recurrence risk in diabetic foot ulcers

Transepidermal water loss (TEWL) quantifies passive water diffusion through the epidermis and is widely used as a noninvasive indicator of permeability barrier integrity. Its use as a clinical or research endpoint requires protocol standardization because values may be influenced by temperature, humidity, airflow, anatomical site, device type, probe handling, recent washing, topical product use, and skin temperature (25, 26). Experimental validation studies support TEWL as a marker of graded barrier disruption and recovery (27).

In DFU care, the most direct evidence comes from the NIDDK Diabetic Foot Consortium TEWL Study. In this multicenter prospective cohort, elevated TEWL at recently closed DFU sites was associated with increased ulcer recurrence, linking local barrier recovery at the healed ulcer site to recurrence vulnerability (7). A related analysis of early maintenance of DFU closure found that higher TEWL at the closed wound site marked compromised functional closure and was associated with recurrence (28). Similarly, an exploratory study of clinically closed chronic wounds showed that elevated TEWL after closure predicted subsequent wound recurrence (6).

These findings support TEWL as a candidate marker of incomplete functional closure, but they do not make it a standalone clinical standard. Absolute values may vary across devices, protocols, anatomical sites, and patient populations, and universally accepted thresholds for post-DFU closure care have not yet been established. Where possible, TEWL should be interpreted with contralateral or periwound comparison, standardized skin assessment, xerosis and fissure scoring, callus evaluation, plantar pressure, and other recurrence-risk factors (25, 26). TEWL should therefore be regarded as one measurable dimension of functional closure rather than a surrogate endpoint that fully captures functional closure on its own.

Within this framework, TEWL has three potential roles: assessing functional closure after apparent healing, enriching recurrence-prevention trials by identifying barrier-deficient healed sites, and serving as a mechanistic or intermediate outcome in studies of barrier-repair interventions. Further studies are needed to define reproducible thresholds, test device comparability, and determine whether TEWL-guided management can reduce recurrence. Candidate indicators for post-closure recurrence-risk assessment are summarized in **Table 2**.

**Table 2:** Candidate indicators for post-closure recurrence-risk assessment after diabetic foot ulcer healing.

Indicator	What it reflects	Advantages	Limitations	Representative references
Transepidermal water loss (TEWL)	Epidermal permeability barrier integrity and residual water-barrier leakage at the healed wound site	Objective, noninvasive, quantitative, and directly related to skin barrier function; may identify clinically closed but functionally vulnerable wound sites	Requires dedicated equipment; sensitive to temperature, humidity, airflow, anatomical site, device type, recent washing, topical product use, and probe handling; clinically actionable thresholds may vary across devices and populations.	(6, 7, 25–28)
Stratum corneum hydration	Water content and hydration status of the superficial stratum corneum	Generally less expensive and more accessible than TEWL; useful for monitoring xerosis and response to moisturizers or barrier-repair products	Does not directly measure barrier leakage; influenced by recent cleansing, topical products, ambient conditions, and probe pressure; should be interpreted as complementary to TEWL rather than a substitute.	(11, 13, 19, 20, 26, 29–38)
Xerosis, fissure, callus, and scar-fragility scoring	Visible clinical manifestations of impaired hydration, barrier fragility, mechanical stress, and pre-ulcerative skin vulnerability	Low cost, clinically practical, feasible in resource-limited settings, and directly relevant to routine foot examination	Semi-quantitative and observer-dependent; requires training and scoring standardization; may not detect subclinical barrier dysfunction before visible skin changes appear.	(14–20, 29, 36, 39)
Standardized foot photography	Longitudinal visual documentation of the healed site, periwound skin, fissures, callus, erythema, maceration, and early skin breakdown	Low cost, reproducible when standardized, useful for blinded adjudication and follow-up comparison, and feasible in outpatient or remote-monitoring settings	Image quality depends on lighting, distance, angle, scale calibration, and operator technique; photographs do not directly measure barrier function or mechanical load.	(8, 9, 40–42)
Plantar pressure assessment	Mechanical loading, repetitive stress, and pressure concentration at healed or high-risk plantar sites	Helps explain site-specific recurrence; useful for guiding footwear modification, offloading, callus control, and pressure redistribution	Requires pressure platform or in-shoe measurement devices; results may vary with gait, footwear, sensor type, and testing protocol; not a direct measure of skin barrier recovery.	(2, 39, 41, 43)
Plantar temperature monitoring	Local inflammation, repetitive tissue stress, thermal asymmetry, and potential pre-ulcerative injury	Can support home-based or remote monitoring; may detect inflammatory or pressure-related tissue stress before visible skin breakdown	Patient adherence and behavioral response are critical; single daily measurements may miss temporal variability; device validation and workflow integration remain important challenges.	(41–49)

DFU, diabetic foot ulcer; TEWL, transepidermal water loss. These indicators should be considered complementary rather than interchangeable. TEWL most directly reflects epidermal water-barrier integrity, whereas stratum corneum hydration, clinical skin scoring, standardized photography, plantar pressure assessment, and plantar temperature monitoring provide additional information on skin condition, mechanical loading, inflammatory stress, and longitudinal surveillance.

## Strength of evidence and translational distance

The evidence supporting functional closure differs in its proximity to healed DFU recurrence. This distinction is important because the proposed framework draws on direct recurrence data, biophysical studies of diabetic skin, and intervention studies of foot xerosis. These evidence streams are complementary, but they should not be interpreted as equivalent.

In the revised framework, we distinguish three levels of inference: established evidence, emerging DFU-specific evidence, and hypothesis-driven extrapolation. Established evidence includes high recurrence rates after healing and the need for continued prevention. Emerging evidence includes the association between elevated TEWL at recently closed DFU sites and recurrence. Hypothesis-driven extrapolation includes the proposed causal sequence in which barrier leakiness promotes microbial or irritant penetration, innate immune activation, and recurrent breakdown. These distinctions are important because the current literature supports functional closure as a research framework, not yet as a consensus clinical endpoint.

The most direct evidence comes from DFU-specific recurrence studies and prevention guidance. Recurrence cohorts and systematic reviews show that a healed DFU remains a high-risk condition, and IWGDF guidance supports continued prevention, surveillance, footwear optimization, treatment of pre-ulcerative lesions, and professional foot care after healing (1–5). Within this direct evidence tier, the NIDDK Diabetic Foot Consortium TEWL Study and related DFU analyses are especially relevant because they measured TEWL at recently closed DFU sites and linked higher values with recurrence or compromised maintenance of closure (7, 28).

A second tier consists of indirect diabetic skin evidence. Studies of people with diabetes have reported reduced stratum corneum hydration, altered permeability barrier function, xerosis, fissuring, altered skin mechanics, and associations with neuropathy or sudomotor dysfunction (11–20). These findings support the biological plausibility that a newly closed ulcer may remain fragile in a metabolically and neurologically compromised cutaneous environment. However, many of these studies examined non-ulcerated diabetic skin or broader diabetic populations. They therefore support mechanism and risk interpretation, but they do not by themselves prove that barrier impairment at a healed DFU site causes recurrence.

A third and more translationally distant tier comes from barrier-repair and moisturizer studies. Urea-, lactic acid-, glycerin-, occlusive-, and lipid-supporting formulations can improve xerosis, hydration, and visible foot skin condition (29–38). These outcomes are clinically relevant, particularly when fissuring or dryness is present. Nevertheless, most available studies were not performed specifically in recently healed DFU sites and were not designed or powered to test recurrent ulceration. The current argument should therefore not be read as evidence that moisturization alone prevents DFU recurrence. Rather, it provides a rationale for targeted trials in patients with demonstrable post-closure barrier vulnerability.

## Foot skin care and barrier-repair interventions: evidence and remaining gaps

Against this evidence background, barrier-repair interventions should be discussed with both interest and caution. If post-closure vulnerability is partly related to impaired skin barrier recovery, targeted skin care may become a useful adjunct to diabetic foot ulcer recurrence prevention. In routine practice, foot skin care is already recommended for people with diabetes, particularly when dry skin, fissuring, callus, or a previous ulcer is present. However, evidence supporting specific products, application protocols, and recurrence-related outcomes remains limited. A recent scoping review identified only a small number of interventional studies of foot skin care for dry skin in people with diabetes, and most assessed xerosis, hydration, fissures, or skin-condition scores rather than ulcer incidence, recurrence, infection, hospitalization, or amputation (29).

The biological rationale for moisturizing and barrier-repair therapy is nevertheless strong. Dry diabetic foot skin is prone to scaling, roughness, fissuring, and superficial cracking. These changes may compromise the physical barrier, increase susceptibility to irritation, and create potential portals of entry for microorganisms. In patients with neuropathy, minor fissures or skin breaks may progress unnoticed under repetitive pressure or friction. Interventions that improve stratum corneum hydration, soften hyperkeratotic skin, reduce fissuring, and support barrier function may therefore reduce pre-ulcerative skin injury. However, biological plausibility alone is not sufficient; what remains necessary is evidence that improving skin condition leads to fewer recurrent ulcers.

Early clinical studies demonstrated that topical moisturizers can improve xerosis of the feet in patients with diabetes. In a prospective, randomized, controlled, double-blind study, regular use of a moisturizer was beneficial for moderate-to-severe xerosis of the feet. The test moisturizer contained 10% urea and 4% lactic acid and was compared with its vehicle base. This study supported the practical observation that daily topical therapy can improve dry diabetic foot skin and helped establish urea- and lactic acid–containing formulations as reasonable candidates for foot skin care interventions. However, the outcomes were focused on xerosis improvement rather than DFU occurrence or recurrence (30).

Additional early work evaluated moisturizer use in the foot care of patients with diabetes. The Pédimed^®^ study provided further clinical support for the use of topical moisturizing care in diabetic feet and contributed to the view that dryness and fissuring are modifiable skin conditions. These studies are relevant because they demonstrate feasibility, tolerability, and improvement in visible skin condition. However, they should be interpreted cautiously in the context of recurrence prevention, because they were not designed to determine whether moisturizing therapy reduces recurrent ulceration or other hard diabetic foot outcomes (31).

Subsequent studies extended this evidence by evaluating more specialized emollient formulations. A randomized, evaluator-blinded controlled trial showed that a cream containing urea, arginine, and carnosine improved skin hydration and reduced dryness in patients with type 2 diabetes compared with a glycerol-based emollient product. A later 8-month assessor-blinded controlled trial suggested that this formulation produced sustained improvement in severe foot xerosis compared with a standard glycerol-based emollient cream, with measurable benefit observed early during treatment. These findings are clinically relevant because they indicate that barrier-oriented topical therapy can produce sustained improvements in foot skin hydration and dryness in patients with diabetes (32, 33).

Other randomized trials have reached similar conclusions using different emollient formulations. In a double-blind, randomized, vehicle-controlled clinical trial, regular use of an emollient cream significantly improved xerosis in the diabetic foot, with improvements in xerosis assessment scores, overall skin score, hydration index, roughness measures, desquamation-related assessment, and patient-reported outcomes. More recent trial activity has continued to compare pharmaceutical skin care creams for xerotic feet in persons with diabetes, using outcomes such as dryness severity, skin hydration, and visible skin condition. Together, these studies support the concept that topical emollients and barrier-supporting products can improve the condition of diabetic foot skin, but they do not yet establish prevention of recurrent DFU (34, 35).

The broader foot xerosis literature is consistent with these findings. A systematic review of moisturizers for the treatment of foot xerosis concluded that several topical formulations may improve dryness-related outcomes, and that urea-containing treatments are among the most frequently studied. However, the review also emphasized heterogeneity in study quality, methodology, formulations, outcome measures, and follow-up duration, which limits firm conclusions about the superiority of one active ingredient over another. This limitation is important for DFU research because improvement in xerosis is a surrogate skin outcome, whereas recurrent ulceration is a harder and more clinically consequential endpoint (36).

The choice of active ingredients in future barrier-repair studies should be biologically informed. Urea can act as a humectant and, depending on concentration, may also soften hyperkeratotic skin and support desquamation. Lactic acid and related alpha-hydroxy acids can support hydration and mild keratolytic activity. Glycerin attracts and retains water in the stratum corneum. Occlusive agents such as petrolatum, liquid paraffin, and soft paraffin reduce evaporative water loss by forming a surface film. Lipid-repair formulations containing ceramides, cholesterol, or fatty acids may theoretically support restoration of the stratum corneum lipid matrix. For post-DFU closure care, the optimal formulation is likely to require a balance between hydration, barrier reinforcement, tolerability, low irritation potential, and safety on fragile healed skin (37, 38).

The selection of barrier-repair formulations for post-closure DFU care should be guided by the dominant skin phenotype, the intended barrier-related action, and the available level of evidence. Several ingredients, including urea, glycerin, lactic acid, occlusive agents, and lipid-supporting components, have been used to improve xerosis, stratum corneum hydration, or visible skin condition. However, direct evidence that any specific ingredient or formulation prevents recurrent DFU remains limited. **Table 3** summarizes candidate barrier-repair ingredients, their potential biological roles, plausible post-closure clinical scenarios, and the current evidence base.

**Table 3:** Barrier-repair ingredients and their potential relevance after diabetic foot ulcer closure.

Ingredient	Main barrier-related actions	Potential clinical scenario after DFU closure	Evidence and notes
Urea	Acts as a humectant; improves stratum corneum hydration; softens hyperkeratotic skin; at higher concentrations may exert keratolytic effects	Dry plantar skin, hyperkeratosis, callus-prone areas, and xerotic skin around a healed ulcer site	Evidence: improves xerosis and hydration in diabetic foot xerosis or foot xerosis studies; recurrence endpoints remain insufficiently tested. Immunological/inflammatory relevance: likely indirect, through reduced hyperkeratosis, fissuring, irritant entry, and microbial access rather than proven immune modulation (23, 30, 32, 33, 36, 37).
Glycerin	Humectant; attracts and retains water in the stratum corneum; improves skin softness and hydration	General dry foot skin, mild xerosis, and maintenance therapy after improvement of severe dryness	Evidence: used as comparator or active moisturizer in diabetic foot xerosis trials; recurrent DFU prevention has not been demonstrated. Immunological/inflammatory relevance: mainly indirect through hydration and reduced irritant exposure; DFU-specific immune outcome data are lacking (23, 32, 33, 36, 38).
Lactic acid	Humectant and mild keratolytic; supports hydration and desquamation regulation; may reduce scaling	Dry, scaly, or mildly hyperkeratotic plantar skin; xerosis with desquamation	Evidence: a 10% urea/4% lactic acid moisturizer improved moderate-to-severe foot xerosis compared with vehicle, but recurrence was not tested. Immunological/inflammatory relevance: may reduce scaling and fissure-prone desquamation; excessive keratolysis should be avoided on fragile healed skin (23, 30, 36).
Ceramides	Support restoration of the stratum corneum lipid matrix; may improve permeability barrier function when incorporated into lipid-repair formulations	Barrier-fragile skin, low skin hydration, impaired barrier recovery, or repeated dryness and irritation around a healed DFU site	Evidence: biologically plausible for lipid-matrix restoration, but post-DFU closure data remain limited. Immunological/inflammatory relevance: restoration of lamellar lipid organization may reduce barrier-driven inflammatory signaling; DFU-specific proof is lacking (12, 23, 24, 38).
Dimethicone	Silicone-based occlusive and protective agent; forms a surface film; may reduce friction and evaporative water loss	Areas exposed to repetitive friction, shoe-related rubbing, fragile scar skin, or periwound skin requiring low-irritation protection	Evidence: low-irritation silicone protectant with potential value in friction-prone or fragile skin; DFU recurrence-prevention evidence is limited. Immunological/inflammatory relevance: expected to be indirect through reduced friction, maceration, and irritant exposure (23, 38).
Petrolatum/liquid paraffin/soft paraffin	Occlusive agents; reduce evaporative water loss; improve softness and barrier support by forming a protective surface film	Dry fissured skin, plantar xerosis, maintenance barrier care, and protection of fragile healed skin from external irritants	Evidence: may reduce water loss and protect dry fissured skin; hard DFU outcomes require study. Immunological/inflammatory relevance: barrier stabilization may reduce external irritant penetration, but direct immune modulation has not been established (23, 24, 29, 36, 38).

DFU, diabetic foot ulcer. These ingredients have evidence or biological plausibility for improving xerosis, hydration, barrier support, or skin protection. However, direct evidence that any specific ingredient prevents recurrent DFU remains limited. Future trials should evaluate these formulations using recurrence-related clinical endpoints together with intermediate barrier outcomes such as TEWL, stratum corneum hydration, fissure burden, and callus severity.

The most important limitation of the current evidence is the nature of the endpoints. Most studies have evaluated xerosis scores, clinical skin appearance, skin hydration, roughness, desquamation, fissures, or patient perception. These endpoints are useful and biologically meaningful, but they do not establish whether barrier-repair therapy prevents clinically important events. Few studies have been designed to determine whether topical skin care reduces DFU incidence, recurrent ulceration, infection, hospitalization, need for debridement, or amputation. As a result, the field has evidence that barrier-directed products can improve skin condition, but much less evidence that they improve durable diabetic foot outcomes (29, 36).

This distinction is particularly important in the post-closure setting. A recently healed DFU represents a high-risk state in which the prevention target is not simply dry skin, but recurrent tissue breakdown. If elevated TEWL identifies a healed site with incomplete barrier recovery, barrier-repair therapy could be tested in a biologically enriched population. Such a trial would differ from previous foot skin care studies in several ways: it would enroll patients after DFU closure, select or stratify patients according to barrier vulnerability, use recurrent ulceration as the primary outcome, and measure changes in TEWL or skin hydration as mechanistic secondary endpoints.

For clinical translation, safety and usability are essential. A barrier-repair product intended for recently healed DFU sites should avoid excessive irritation, maceration, and occlusion in interdigital spaces. It should be compatible with therapeutic footwear and offloading devices, easy for patients or caregivers to apply, and safe for long-term use. Trials should also standardize application frequency, anatomical application sites, foot cleansing procedures, avoidance of application to open wounds or infected areas, and adherence monitoring. Without these details, it will be difficult to distinguish failure of the intervention from poor implementation.

Overall, current evidence supports the feasibility and biological plausibility of foot skin care and barrier-repair interventions in patients with diabetes. Yet the field has not fully moved from treating xerosis to preventing ulcer recurrence. The next step is to test whether targeted barrier repair in patients with recently closed DFUs can improve durable healing. Such studies should incorporate hard clinical endpoints, including recurrent ulceration, time to recurrence, infection, re-hospitalization, and amputation, while also measuring intermediate barrier outcomes such as TEWL, stratum corneum hydration, fissuring, and callus burden. This transition from dermatologic improvement to recurrence prevention represents a key opportunity for future DFU research.

## Discussion and future directions: recurrence-prevention trials and precision post-closure management

Future DFU recurrence-prevention trials should learn from evidence showing that a simple, well-tolerated intervention is not necessarily sufficient to reduce clinically meaningful foot outcomes. In a randomized, double-blind, soap-and-water controlled clinical trial, daily use of 2% chlorhexidine wipes for foot washing over 1 year did not significantly reduce new foot complications compared with soap-and-water wipes (40). The trial is instructive: daily foot-directed care can be implemented, but antimicrobial cleansing alone may not address the dominant biological and biomechanical pathways leading to ulceration.

Risk enrichment is particularly important in the post-closure DFU population. Patients with a recently healed DFU are already at high risk, but this group remains heterogeneous. Some patients may be primarily vulnerable because of incomplete barrier recovery, whereas others may recur because of high plantar pressure, callus, deformity, ischemia, infection susceptibility, or poor adherence to offloading. Guideline-based prevention should remain the foundation of any recurrence-prevention trial (2). Barrier-repair interventions should be tested as an addition to, rather than a replacement for, established preventive care.

A precision post-closure framework would classify patients according to their dominant recurrence phenotype. A barrier-vulnerable phenotype may be characterized by high TEWL, xerosis, fissuring, fragile scar skin, or low stratum corneum hydration. A pressure-dominant phenotype may be characterized by elevated plantar pressure, recurrent callus, deformity, limited joint mobility, or poor footwear fit. An inflammation- or infection-prone phenotype may be characterized by local temperature elevation, maceration, interdigital fungal disease, recurrent superficial skin breaks, or a history of soft-tissue infection. This phenotype-based approach may help match the intervention to the mechanism most likely to drive recurrence in an individual patient.

Plantar pressure and temperature monitoring are complementary to barrier assessment rather than substitutes for it. Plantar pressure measurement helps explain site-specific recurrence and can guide footwear modification, offloading, callus control, and pressure redistribution (39). Temperature monitoring may detect inflammation or repetitive tissue stress before visible breakdown, but randomized evidence is mixed; benefit depends on adherence, the patient response to abnormal readings, and integration into clinical workflows (44–47). Digital foot health systems that combine pressure, temperature, photography, patient feedback, and clinician alerts are promising, but clinical translation still requires device validation, usability testing, actionable thresholds, and workflow integration (41–43, 48–55).

Several implementation barriers must be considered before the proposed framework can be translated into routine care. TEWL measurement requires standardized room temperature and humidity, acclimatization time, consistent anatomical mapping, avoidance of recent washing or topical products, and trained operators. Device variability, calibration, probe pressure, cost, appointment time, reimbursement, staff training, and integration with existing diabetic foot pathways may limit broad adoption. For this reason, the framework should allow a tiered approach: TEWL and hydration testing in instrumented centers, and standardized skin scoring, photography, callus assessment, and clinical surveillance in clinics without biophysical devices.

Recent publications reinforce the need to distinguish visible healing from durable post-healing care. The IWGDF practical guideline update emphasizes prevention, classification, management, and organized levels of diabetic foot care across the disease course (56). A post-healing transition review similarly argues that the weeks after DFU closure represent a distinct phase in which residual tissue mechanics and offloading remain clinically important (57). Three-year recurrence data and multidisciplinary follow-up studies further support prolonged remission-focused surveillance after closure (58, 59). Risk-prediction models are also developing, but remain heterogeneous and require external validation before routine implementation (60, 61).

This validation gap is not unique to DFU recurrence. Prognostic modeling in other tissue-repair settings shows the same methodological bottleneck. A 2026 systematic review and meta-analysis of prediction models for delayed union and nonunion after fracture identified frequent reliance on apparent performance, limited independent external validation, sparse calibration reporting, substantial heterogeneity, and high risk of bias, with model performance generally attenuating under validation (62). This parallel supports a cautious approach to DFU recurrence algorithms: risk models should not be implemented merely because development-stage discrimination appears promising, but should undergo external validation, calibration assessment, and evaluation of clinical utility in independent post-healing populations.

For future randomized trials, the most important design question is not simply which product or technology should be tested, but which patient subgroup should be enrolled. A trial of barrier-repair therapy should ideally enroll patients with recently closed DFUs who show evidence of barrier vulnerability, such as elevated TEWL, low stratum corneum hydration, xerosis, fissuring, or fragile scar skin. All participants should receive standard preventive care, including offloading assessment, footwear optimization, callus management, and education. The primary outcome should be recurrent ulceration or time to recurrence, while secondary outcomes should include TEWL, skin hydration, fissuring, callus burden, infection, re-hospitalization, amputation, adherence, and adverse skin reactions.

Future trials of barrier-repair therapy after DFU closure should therefore test a biologically plausible intervention in an appropriately enriched high-risk population. The key design challenge is not only product selection, but also target-population definition, risk enrichment, background standard care, and clinically meaningful endpoints. **Table 4** summarizes proposed design elements for randomized controlled trials evaluating barrier-repair therapy as an adjunct to established recurrence-prevention care after DFU healing.

**Table 4:** Core design elements for future randomized trials of barrier-repair therapy after diabetic foot ulcer closure.

Design element	Recommended approach	Rationale and key considerations	Representative references
Target population	Patients with recently healed diabetic foot ulcers, preferably enrolled within 2–12 weeks after complete epithelial closure	Recently healed DFU sites remain highly vulnerable to recurrence. The healed ulcer site should be clearly identifiable and clinically closed at baseline.	(1–7, 63, 64)
Risk enrichment	Enroll or stratify patients with elevated TEWL, low stratum corneum hydration, xerosis, fissuring, fragile scar skin, or recurrent callus	Barrier-repair therapy is most biologically plausible in patients with incomplete functional closure or visible skin-barrier vulnerability.	(6, 7, 11, 14–20, 28, 29, 36)
Intervention	Standardized barrier-repair formulation applied to the healed ulcer site and surrounding high-risk skin	Candidate formulations may include humectants, occlusives, keratolytic-hydrating agents, or lipid-repair components with low irritation potential.	(29–38)
Control	Matching vehicle or base cream with similar appearance, texture, odor, packaging, and application schedule	A matched control helps preserve blinding and separates active barrier-repair effects from nonspecific moisturizing or trial participation effects.	(30, 34, 40)
Background standard care	All participants should receive guideline-based recurrence-prevention care, including education, foot inspection, callus care, footwear assessment, vascular assessment when indicated, and offloading optimization	Barrier repair should be tested as an adjunct to established DFU prevention, not as a substitute for pressure reduction, footwear optimization, or professional foot care.	(2, 39)
Primary outcome	Recurrent diabetic foot ulcer within 12 months, or time to first recurrent ulcer	Recurrent ulceration is the most clinically meaningful endpoint for post-closure prevention trials. Time-to-event analysis captures both recurrence and timing.	(1–7, 63, 64)
Secondary and mechanistic outcomes	Original-site recurrence, new-site ulceration, foot infection, hospitalization, amputation, adverse skin reactions, TEWL, stratum corneum hydration, xerosis score, fissure score, callus burden, standardized photographs, and patient-reported skin symptoms	These outcomes assess both clinical benefit and whether the intervention improves barrier-related intermediate endpoints.	(1, 2, 6, 7, 11, 13–20, 25–38, 40)
Confounders and adherence	Account for peripheral artery disease, neuropathy severity, prior ulcer history, ulcer location, plantar pressure, deformity, callus, footwear, kidney disease, glycemic status, infection history, topical product adherence, and offloading adherence	These factors strongly influence recurrence risk and should be balanced, stratified where appropriate, adjusted in analyses, and monitored throughout follow-up.	(1, 2, 15–18, 39, 40, 44–47, 49, 63, 64)

DFU, diabetic foot ulcer; TEWL, transepidermal water loss. Future trials should distinguish improvement in intermediate skin-barrier outcomes from reduction in clinically meaningful recurrence-related endpoints. Barrier-repair therapy should be evaluated as an adjunct to established preventive care, including offloading, footwear optimization, vascular assessment, infection surveillance, and professional foot care.

Where TEWL instruments are not available, pragmatic alternatives may still be valuable. Standardized clinical skin scores, digital photographs, assessment of xerosis and fissures, callus grading, and low-cost measures of stratum corneum hydration could be used for risk stratification or as secondary outcomes. These tools are less specific than TEWL for barrier integrity, but they may improve feasibility in resource-limited settings. A practical trial design could therefore include two tiers: an instrumented subgroup with TEWL and hydration testing, and a broader pragmatic cohort using standardized clinical assessment and photography.

**Figure 3:**
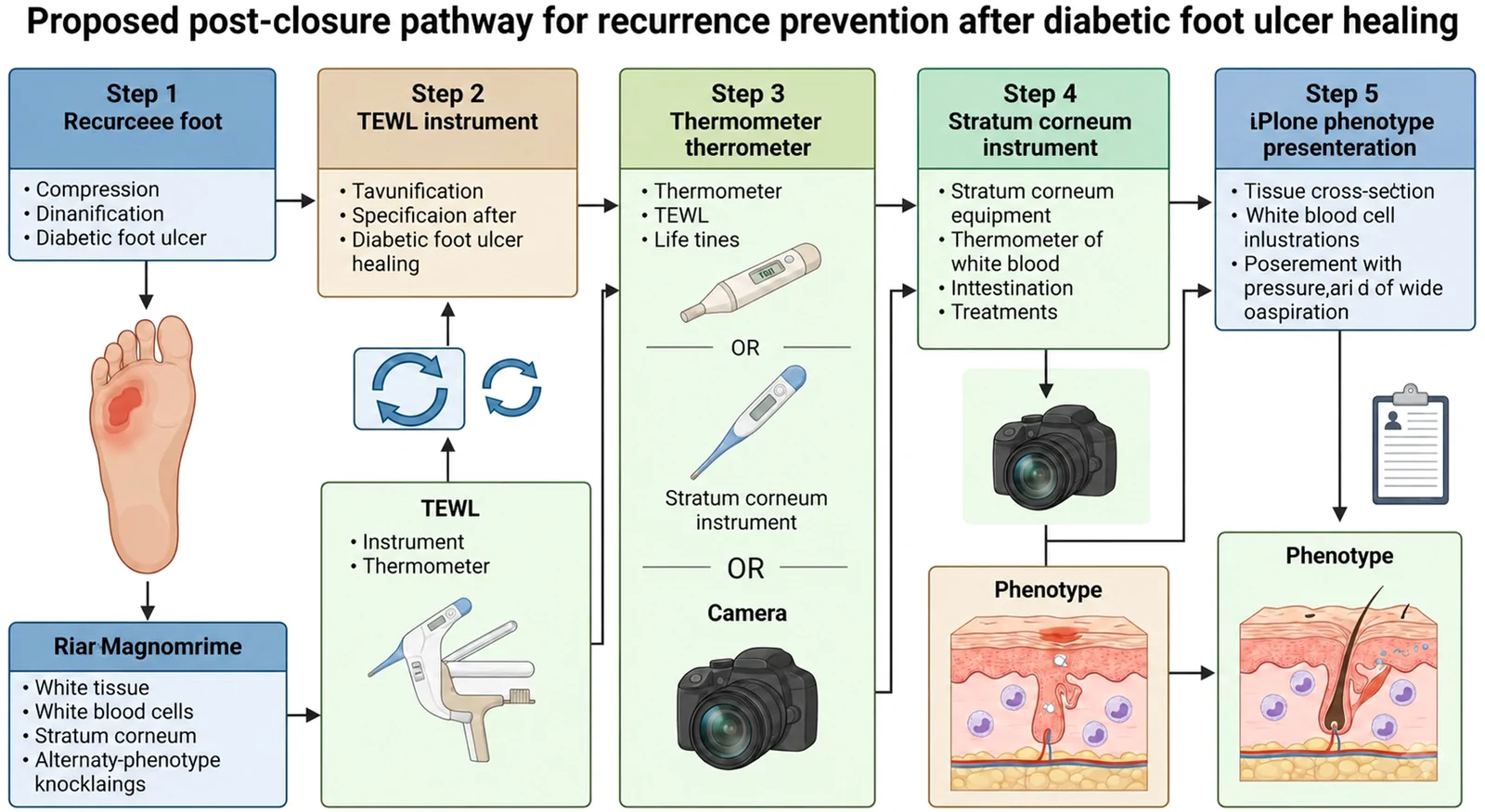
Proposed post-closure pathway for recurrence prevention after diabetic foot ulcer healing. After apparent diabetic foot ulcer (DFU) closure, an early post-closure assessment should be performed, preferably within 2 weeks, to identify residual vulnerability at the healed site and surrounding foot skin. Instrumented assessment may include transepidermal water loss (TEWL), stratum corneum hydration, plantar pressure, and plantar temperature. When these tools are unavailable, pragmatic assessment may include clinical skin health scoring, xerosis and fissure assessment, callus grading, and standardized foot photography. Based on these findings, patients may be stratified into barrier-vulnerable, pressure-dominant, or inflammation/infection-prone phenotypes. Low/moderate-risk patients may continue routine barrier care, offloading optimization, regular self-monitoring, and follow-up every 3–6 months. High-risk patients may require intensified barrier-repair therapy, customized offloading footwear, more frequent professional foot care, and follow-up every 1–2 months. This pathway emphasizes that DFU management should not end at visible closure, but should transition into structured long-term monitoring and maintenance of durable closure. The pathway is presented as a conceptual framework for research and multidisciplinary discussion; it requires prospective validation before routine clinical implementation.

A practical post-closure pathway should translate the concept of functional closure into a structured clinical workflow. After DFU closure is confirmed, patients may undergo early reassessment, preferably within 2 weeks, to evaluate skin barrier status, local skin quality, standardized foot photographs, and mechanical loading when available. Patients with lower-risk profiles may continue standard barrier care, offloading optimization, self-monitoring, and routine follow-up, whereas patients with high-risk features may require intensified barrier therapy, customized offloading footwear, more frequent professional foot care, and closer surveillance. This proposed post-closure management pathway is summarized in **Figure 3**. This pathway is intended as a conceptual model for future validation and should not be interpreted as an evidence-based clinical algorithm or practice guideline.

The goal of precision post-closure management is not to replace existing DFU prevention strategies, but to make them more biologically and clinically targeted. A patient with high plantar pressure may benefit most from pressure-based footwear modification and callus control. A patient with temperature elevation may require intensified monitoring for inflammation or impending tissue breakdown. A patient with high TEWL or marked xerosis may require barrier-repair therapy and closer inspection of the healed site. Many patients will have overlapping phenotypes and require combined interventions. Recognizing these differences may explain why single, uniform interventions often fail to produce large effects in unselected high-risk populations.

In summary, the next generation of DFU recurrence-prevention studies should move from generalized foot care toward risk-enriched and phenotype-guided post-closure management. TEWL and other measures of skin barrier quality offer a way to identify patients with incomplete functional closure. These measures should be integrated with established assessments of plantar pressure, callus, footwear, vascular status, temperature, infection risk, and adherence. Such an approach may allow future trials to test barrier-repair strategies in the patients most likely to benefit and may ultimately shift DFU care from reactive treatment of recurrent ulcers to proactive maintenance of durable closure.

## Limitations of this review

Several limitations should be acknowledged. First, the evidence base supporting this framework is uneven. Although emerging DFU-specific data link elevated TEWL with impaired maintenance of closure, the available evidence remains limited and should be regarded as hypothesis-generating rather than definitive. Second, much of the biological rationale comes from studies of diabetic skin barrier dysfunction rather than from healed DFU tissue itself. Third, direct clinical evidence that barrier-repair interventions reduce recurrent DFU is still insufficient; most studies have focused on xerosis, hydration, fissuring, or skin-condition scores rather than hard recurrence-related outcomes. Fourth, TEWL measurement is sensitive to device type, anatomical site, environmental conditions, probe handling, recent cleansing, and topical product use, and clinically actionable thresholds for healed DFU sites have not yet been standardized. Finally, this article is a narrative review rather than a systematic review or meta-analysis. The proposed framework should therefore be interpreted as a clinically oriented synthesis intended to guide future research, not as a definitive practice guideline. In addition, functional closure has not yet been prospectively validated as an independent predictor of DFU recurrence or endorsed as a consensus endpoint in diabetic foot care. The proposed pathway also requires feasibility testing with respect to TEWL reproducibility, device cost, clinician training, and workflow integration in routine clinics.

## Conclusions

Visible closure remains an essential milestone in DFU care, but it should not be treated as evidence that the healed site has fully recovered. The high frequency of recurrence after apparent healing suggests that some ulcers close morphologically while remaining biologically fragile. Functional closure offers a more clinically useful framework by asking whether the regenerated surface has restored sufficient barrier competence to withstand daily mechanical stress, maintain hydration, limit water loss, and resist microbial entry. TEWL is currently the most direct candidate marker for this concept, although it should be interpreted alongside clinical skin quality, plantar pressure, temperature, vascular status, neuropathy, and offloading adherence. Existing foot skin care studies support improvement in xerosis and hydration, but they have not yet shown that barrier repair prevents recurrent ulceration. The next step is therefore not simply to recommend more moisturization, but to test targeted barrier-repair strategies in patients with demonstrable post-closure vulnerability. Such an approach may help shift DFU management from treating recurrent breakdown toward maintaining durable remission. Accordingly, functional closure should be framed as a barrier-competent and immunologically competent endpoint, rather than as a purely mechanical or hydration-based endpoint. The term should therefore be used cautiously at present: it is a proposed conceptual and research endpoint rather than an established clinical standard.
